# High Tibial Osteotomy for Knee Osteoarthritis with Genu Varum: A Retrospective, Observational Study

**DOI:** 10.3390/jfmk11010129

**Published:** 2026-03-23

**Authors:** Ana Ramos, Jordi Zafra, Jordi Villalba

**Affiliations:** 1 Facultad de Medicina, Universitat de Vic, Universitat Central de Catalunya, Ctra. De Roda, 70, 08500 Vic, Barcelona, Spain; 2 Department of Orthopaedics and Traumatology, Consorci Hospitalari de Vic, Carrer de Francesc Pla el Vigatà, 1, 08500 Vic, Barcelona, Spain

**Keywords:** high tibial osteotomy, knee osteoarthritis, joint preservation, total knee arthroplasty

## Abstract

**Background:** High tibial osteotomy (HTO) is a joint-preserving alternative for patients with medial compartment knee osteoarthritis (KOA), although its use has declined in recent decades in favor of total knee arthroplasty (TKA). This study aimed to evaluate HTO outcomes in patients treated at a tertiary center over the past decade. **Methods:** We conducted a single-center, retrospective, observational cohort study of patients with medial femorotibial KOA and genu varum who underwent HTO. Failure was defined as conversion to TKA. A comparative analysis regarding HTO survival was conducted with preoperative variables and KOA stages in the follow-ups up to 5 years. Univariate and multivariate Cox regression models were built to assess their effect on HTO survival time. The Kaplan–Meier method was used to estimate overall and subgroup survival. Disease progression over time was evaluated with the Bhapkar test. In all cases, *p* < 0.05 was considered statistically significant. **Results:** HTO was successful in 74.6% of the 63 patients. Age was significantly higher in the failure group (*p* = 0.006), and each additional year increased the hazard of failure by 8% (95% CI, 1.01–1.16, *p* = 0.033), although this significance was lost in multivariate analysis (*p* = 0.104). Kaplan–Meier estimated survival was 76.7% at 5 years, with a median survival time of 10.1 years. KOA stage progression was significant 5 years after HTO (*p* < 0.001). **Conclusions:** HTO demonstrated 76.7% survival at 5 years, with radiographic KOA progression over time. The association between age and failure was not maintained after multivariable adjustment.

## 1. Introduction

Knee osteoarthritis (KOA) is the leading cause of disability in older adults, with a prevalence reaching up to 30% in individuals over the age of 65. It is a chronic joint disease characterized by a progressive and complex destructive process of the hyaline cartilage, leading to secondary subchondral bone sclerosis and osteophytosis [1]. Due to its primary symptom, pain, it significantly impairs patients’ quality of life and their ability to perform activities of daily living [2,3,4,5].

The medial femorotibial compartment is the most frequently affected. Under physiological conditions, it supports approximately 55–70% of the load transmitted through the knee during ambulation. This load can increase further in the presence of prior joint injuries or varus malignment, both of which elevate mechanical stress on the compartment and accelerate cartilage degeneration and the onset of KOA [6,7,8]. Similarly, patients often adopt compensatory gait patterns, consciously or unconsciously, to maintain stability or reduce pain. These adaptations can contribute to abnormal biomechanical loading, such as walking with increased toe-out angle or altered joint kinematics throughout the affected limb. In addition, biomechanical markers such as an increased peak external knee adduction moment (KAM) and the presence of varus thrust further increase medial compartment loading and have been linked to disease progression [9,10].

While total knee arthroplasty (TKA) is the standard treatment for advanced KOA, it is generally avoided in the early stages for several reasons, including the desire to preserve bone stock, reduce the risk of future revision surgery, and maintain higher levels of joint proprioception and function [11,12]. Thus, other surgical alternatives that can provide symptomatic relief are preferred. Both unicompartmental knee arthroplasty and high tibial osteotomy (HTO) can reduce pain and postpone the need for TKA. In younger patients, typically under 50 years old, with mild to moderate cartilage degeneration in the medial compartment and in presence of genu varum, HTO is often preferred. By inducing a valgus correction of the mechanical axis, HTO redistributes the load across the joint and realigns the knee, shifting weight from the medial to the lateral compartment, thereby mitigating symptoms [8,13,14,15,16,17].

Uncertainties remain regarding the long-term survival and durability of HTOs. Several factors, such as obesity, preoperative joint damage, and age over 55 years, have been associated with poorer postoperative outcomes. These variables are generally considered negative prognostic indicators, as they are linked to an increased likelihood of requiring conversion to TKA. However, consensus on their role is not clear, and the available literature remains limited [8,13,14,15,18].

The aim of our study was to evaluate the outcomes of HTO performed in a referral center in patients with medial KOA and genu varum over the past decade, and to explore disease progression as well as potential variables associated with treatment success.

## 2. Materials and Methods

This is a single-center, retrospective, observational, cohort study, approved by the Ethics Committee of our hospital (code 25/012, date: 24 February 2025) and conducted in accordance with the Declaration of Helsinki. The Ethics Committee granted a waiver of informed consent, as the study posed no patient risks, involved no new interventions, and relied exclusively on retrospective chart data. All data were anonymized prior to analysis to protect patient confidentiality. Access to the original data was restricted to study investigators within the institution. This study has been reported following the Strengthening the Reporting of Observational Studies in Epidemiology (STROBE) guidelines [19].

Inclusion criteria consisted of patients between 18 and 75 years old, diagnosed with medial femorotibial KOA and genu varum, who had undergone HTO between 2014 and 2024 in the Consorci Hospitalari de Vic (Barcelona). Cases in which complete clinical or radiographic information could not be obtained from medical records, or with less than one year of follow-up, were excluded. In addition, cases in which the planned mechanical correction was not achieved intraoperatively, resulting in postoperative alignment outside the predefined target range (0° ± 3° of mechanical axis), were excluded, as they were considered technical deviations from the planned surgical procedure.

All data were extracted from the patients’ clinical records. We collected anthropometric data (sex, age, and BMI), comorbidities (arterial hypertension, diabetes mellitus, heart failure, respiratory failure, and mental disorders) and the preoperative KOA stage, which was determined by evaluating preoperative radiographs according to the Kellgren–Lawrence radiological scale, using the original discrete grades (0–4) [20]. Discrepancies were resolved by consensus. For clinical stratification and statistical analysis, KL grades were subsequently organized into predefined adjacent categories according to our institutional protocol: normal (grade 0), doubtful (grades 0–1, grade 1), mild (grades 1–2, grade 2), moderate (grades 2–3, grade 3), and severe (grades 3–4, grade 4) [21]. This secondary grouping was performed since in routine practice neighboring KL grades often correspond to transitional radiographic stages with partially overlapping therapeutic implications.

Patients underwent either a lateral closing wedge osteotomy or a medial opening wedge osteotomy performed by a member of the Department of Orthopaedics and Traumatology at our center, each with more than five years of experience in these surgical techniques. Lateral closing wedge osteotomy was performed in the supine position, with the knee on 90° flexion. An incision was made on the lateral aspect of the leg, using the anterior tibial tuberosity and the head of the fibula as reference. The iliotibial tract was removed to access the tibia, while protecting the neurovascular structures. According to surgical planning, cutting points of the osteotomy were marked on the tibia and the subtraction of a triangle-shaped bone wedge was performed, while preserving the medial cortex of the tibia. The two bone surfaces resulting from the cut and subtraction were in contact and were fixed with two osteosynthesis staples. Medial opening wedge osteotomy was performed in the supine position, with a previous arthroscopy performed to evaluate the state of cartilage and meniscal lesions. The operated knee was fully extended, and a longitudinal incision was made between the medial border of the tibial tuberosity and the posterior border of the tibia. The surface of the medial collateral ligament was detached or separated backwards, the goose foot was dissected, and the patellar tendon was separated, always protecting the neurovascular structures. Next, the osteotomy was marked and oriented by means of guides, and a transverse cut was made to the proximal tibia with an oscillating saw. Once the cut was made, the osteotomy site was opened by drawing an open wedge shape and keeping the lateral cortex of the tibia intact, using osteotomes, thus achieving correct alignment. This open wedge was filled with a bone graft, and the osteotomy was fixed with a plate, two proximal cancellous screws and two distal cortical screws.

Postoperative radiographic assessments were conducted at 1, 2, and 5 years in all patients. At follow-up, radiographic evaluations were unavailable for two predefined reasons only: (1) patients who had undergone TKA, in whom further radiographic grading of KOA was not feasible due to the presence of the prosthesis, and (2) patients who had not yet reached the respective follow-up time point at the time of data closure. Therefore, missing radiographic data at later time points were primarily attributable to administrative censoring related to follow-up duration rather than to loss to follow-up or selective attrition. Failure of the HTO was defined as the subsequent need for TKA, and survival time was calculated as the interval between HTO and TKA. Indication for conversion to TKA required radiographic progression together with clinical deterioration characterized by persistent pain and/or functional limitation that the patient considered unacceptable for activities of daily living, as well as failure of conservative management—including rehabilitation physiotherapy and adequate analgesic treatment.

All statistical analyses were carried out using R software (R Foundation for Statistical Computing, Vienna, Austria), version 4.4.3 [22]. Descriptive statistics were calculated for all variables, including measures of central tendency and dispersion. A comparative analysis was conducted for all collected variables, stratifying patients into the success group (i.e., those who did not require TKA) and the failure group (i.e., those who subsequently underwent TKA). Parametric or non-parametric tests were applied depending on whether variables followed a normal distribution, which was assessed using the Shapiro–Wilk test. Categorical variables were analyzed using Fisher’s exact test or the χ^2^ test. A complete-case analysis was performed for all statistical tests. Two Cox regression models (univariate and multivariate) were built to assess the effect of selected preoperative variables (age, sex, BMI, HTO type and preoperative KOA grade) on HTO survival time. Given the limited number of failure events (n = 16) and the potential risk of overfitting associated with including multiple covariates, a parsimonious multivariable approach was subsequently adopted. To improve model stability and maintain an appropriate events-per-variable ratio, only the two variables showing the strongest associations in univariate analysis (age and sex) were included in the final multivariable model. The Kaplan–Meier method was used to estimate overall HTO survival time and to compare survival according to sex, age (< or ≥55 years, which was the median age of our sample and has also been identified as a threshold for TKA conversion [23,24]), HTO type, and preoperative KOA stage. Patients who converted to TKA were treated as failure events in the survival analysis and were therefore not censored. Censoring of remaining patients reflects the routine follow-up schedule and administrative censoring at the time of data closure, rather than loss to follow-up. The impact of KOA stage on HTO outcome (success vs. failure) was assessed by comparing stage distribution between the two groups at each follow-up. In addition, disease progression over time was evaluated by the comparison of the preoperative KOA stage with that observed at 1, 2, and 5 years of follow-up with the Bhapkar test, a generalization of McNemar’s test. For this analysis, TKA was incorporated as the worst outcome category to account for surgical conversion. In all cases, *p* < 0.05 was considered statistically significant.

## 3. Results

A total of 63 patients were recruited for the study, having undergone HTO between 2014 and 2024, with a median follow-up time of 3.8 years (1.4–11.2 years).

The majority of patients (71.4%) were male, with a mean age of 53.3 years. Two thirds of patients (66.7%) presented some comorbidity. All anthropometric, clinical and surgery parameters are summarized in Table 1 for the entire cohort and stratified by HTO survival or failure.

Treatment was successful in 74.6% of cases. Median survival time in the failure group was 3.5 years (2.0–5.3) and median follow-up time in the success group was 5.0 years (2.0–8.0). While age was significantly higher in the failure group (*p* = 0.006), no other variable was found to be associated with HTO success. Age was not significantly associated with preoperative KOA state (*p* = 0.319). Similarly, the Cox regression models did not reveal any significant associations between survival time and the variables under study (Table 2), except for age, where each additional year increased the hazard of failure by 8% in the univariate model. However, this association did not remain statistically significant in the multivariable model (*p* = 0.098). The proportional hazards assumption was assessed using Schoenfeld residuals. The global test was non-significant (*p* = 0.419), and no violations were observed for individual covariates (sex: *p* = 0.199; age *p* = 0.826). Visual inspection of the smoothed residual plots showed no evidence of time-varying coefficient trends, as the smoother lines were approximately flat within the confidence bands (Figure S1).

**Figure 1:**
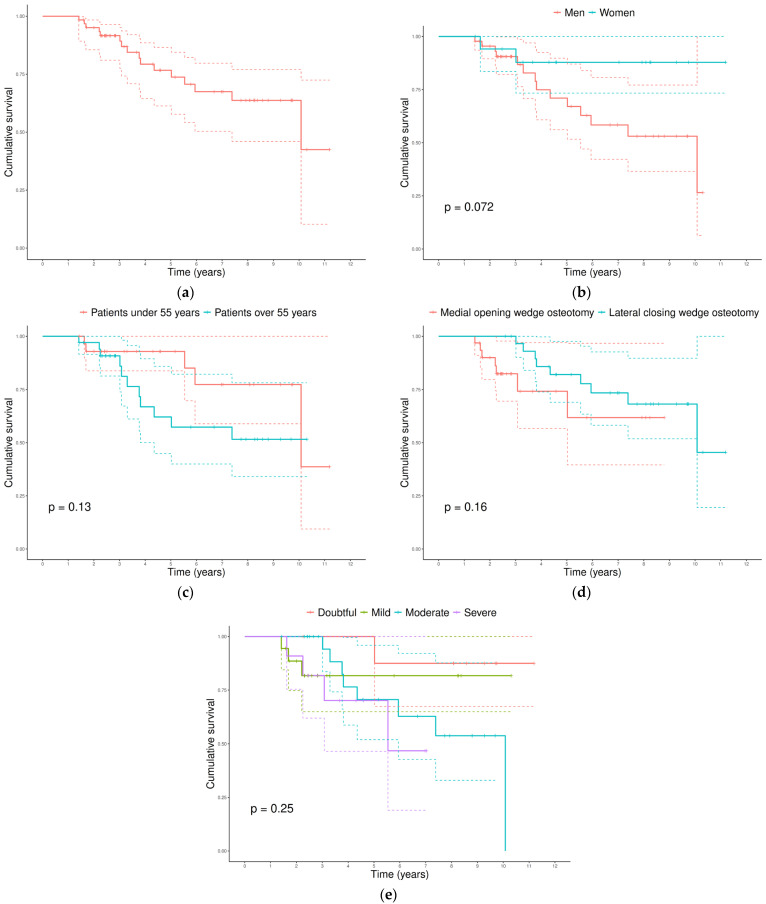
Kaplan–Meier survival curves. (**a**) Overall survival (events = 16). (**b**) By sex (men = 14 events; women = 2 events). (**c**) By age group (<55 years = 5 event; ≥55 years = 11 events). (**d**) By HTO type (medial opening wedge osteotomy = 7 events; lateral closing wedge osteotomy = 9 events). (**e**) By preoperative KOA stage (doubtful = 1 event; mild = 3 events; moderate = 8 events; severe = 4 events). *p*-values for comparisons between groups were calculated using the log-rank test and are shown in the corresponding panels.

The Kaplan–Meier estimated survival rate was 98.4% (95% CI, 95.4–100.0) at 1 year, 95.1% (95% CI, 89.8–100.0) at 2 years, and decreased to 76.7% (95% CI, 65.2–90.2) at 5 years and 63.7% (95% CI, 49.8–81.4) at 10 years (Figure 1a). The median survival time was 10.1 years. Survival estimates according to sex, age, HTO type, and preoperative KOA stage are shown in Figure 1, displaying patterns similar to those observed in the previous analyses. Although *p*-value was close to 0.05 for sex, no statistically significant differences were observed in any of the comparisons after log-rank tests. Detailed survival data for each panel is shown in Figure S2.

The radiological stages of KOA at all study time points are detailed in Table 3, both for the overall patient cohort and stratified according to treatment success. In this regard, no statistically significant differences were observed in the KOA stages of any of the follow-ups between the success and failure groups, indicating that the distribution of KOA stages was comparable between patients who eventually required TKA and those who did not.

**Figure 2:**
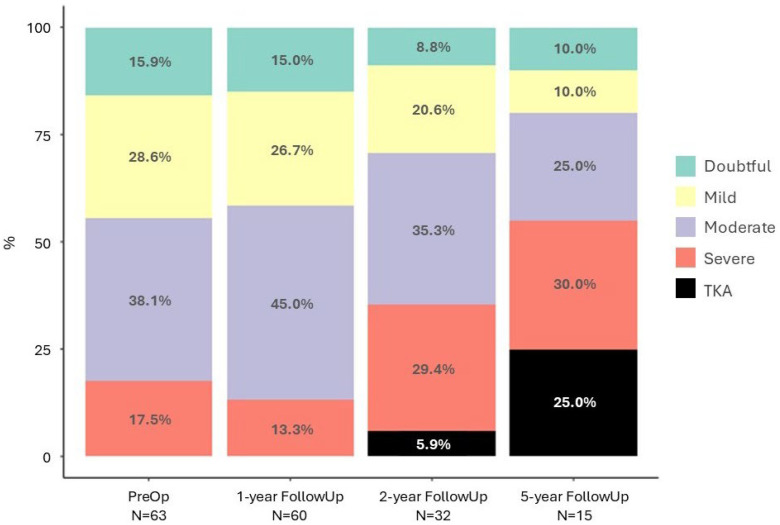
Progression of KOA stage from the preoperative assessment to the 5-year follow-up, including TKAs. The total number of evaluated patients (N) is indicated at each follow-up. Missing data was excluded to improve clarity.

With respect to disease progression, as seen in Figure 2, one year after HTO most patients remained at the same KOA stage as preoperatively (*p* = 0.432). By the second postoperative year, a greater number of patients showed progression, particularly those who had initially presented with a moderate stage, which decreased in favor of the severe category and the occurrence of TKA, although this did not reach statistical significance (*p* = 0.068). At the 5-year follow-up, KOA progression was more pronounced, with the severe stage accounting for 30% of the analyzed patients, and reached statistical significance (*p* < 0.001), indicating a significant long-term trend of pathological deterioration.

## 4. Discussion

The chronic and degenerative nature of KOA implies that there is no definitive cure. Although medical teams generally prefer to exhaust conservative treatments, the transition from pharmacological and rehabilitation strategies to surgical procedures is, in many cases, inevitable given the natural course of the disease [8,25,26,27,28,29,30,31].

While TKA is widely established as the final treatment, with well-documented benefits in symptom reduction and quality of life improvement, the aggressive nature of the procedure is inevitably associated with certain functional limitations that may impact patient satisfaction. Moreover, although TKA survival rates reach around 90% at more than 15 years, the increasing life expectancy and growing number of surgeries performed suggest that the demand for revision arthroplasties will continue to rise in the coming years. Therefore, even though in recent decades surgical decision-making has increasingly favored TKA, leading to a marked decline in the use of HTO, the potential benefits of HTO in terms of joint preservation and delaying prosthetic implantation should not be disregarded [26,27,28,29,30,31,32].

In our study, the Kaplan–Meier estimate of HTO survival at 5 years was 76.7%, and the procedure delayed the need for TKA by a median of 10 years in those who ultimately required it. These results are lower than those reported in previous studies, which ranged from 86% to 100% survival at 5 years [33]. Similarly, the long-term survival estimate was relatively low, indicating that HTO would remain effective in only 63.7% of patients 10 years after surgery. It should be noted that our 10-year estimates lie in the distal portion of the Kaplan–Meier curves, where confidence intervals widen due to the smaller number of patients at risk; therefore, these results should be interpreted with caution. In contrast, Efe et al. (2011) [34] reported an 84% success rate at 10 years, Guarino et al. (2023) [17] a 75.5% rate at 17 years, and Ishizuka et al. (2021) [23] a 75.9% rate at 20 years, which is comparable to our 5-year estimate (76.7%).

These discrepancies may be explained by several factors. First, in our center, HTO was performed in patients with a broader range of KOA stages compared to some of the cited studies—Kellgren–Lawrence grades II-III in Guarino et al. (2023) [17] and grades III-IV in Ishizuka et al. (2021) [23]—due to the different time periods of patient recruitment and the corresponding clinical protocols, which evolve with available evidence. Second, differences in conversion criteria to TKA may partly explain our findings. In our clinical practice, the indication for arthroplasty was not based solely on radiographic progression but required concordant clinical worsening, including sustained pain or functional impairment that patients perceived as incompatible with their daily activities, after insufficient response to non-surgical treatment strategies. Moreover, the decision-making process took into account patient’s expectations and individual tolerance to symptoms within a shared decision-making framework. Such an approach may have favored earlier transition to TKA in some cases when compared with series applying stricter structural criteria. In addition, differences in cultural context and healthcare systems may influence patient’s preferences and acceptance of surgical intervention, and therefore a contextual effect on conversion rates cannot be ruled out [35]. Lastly, our study population may have been too heterogeneous, including variables that might be considered exclusion criteria in other centers. Nonetheless, the main objective of our analysis was to reflect the reality of our clinical practice and to identify factors that could help refine treatment indications, ultimately improving both outcomes and patient satisfaction.

Age was the only variable associated with HTO survival in univariate analysis, suggesting that younger patients at the time of treatment tended to show lower conversion rates to TKA. Aging is naturally associated with reduced bone quality and progressive loss of bone mineral density, which may result in poorer and delayed consolidation, more prone to mechanical failure and microfractures. In addition, KOA stage often correlates with age, with greater joint degeneration observed in older patients, meaning that the benefits of load axis correction may be less pronounced in this group. However, this association did not remain statistically significant in multivariable modeling. The limited number of failure events (n = 16) restricted the statistical power of multivariable analysis and may have contributed to the loss of significance after adjustment. Although previous studies have consistently reported reduced HTO survival with increasing age [16,36,37,38,39,40], the substantial overlap in age distributions between success and failure groups limits our ability to define precise thresholds or establish patient selection criteria based solely on age. Therefore, our findings should be interpreted cautiously as descriptive evidence of an age-related trend rather than as confirmation of age as an independent prognostic factor.

By contrast, although HTO failure was more frequent in men, sex differences were not statistically significant in any of our analyses. The scientific literature has presented contradictory findings regarding sex as a prognostic factor, with some studies reporting higher failure rates in women [38,41], while others found no significant differences between sexes [42,43]. In our case, this result may be explained by stricter preoperative selection criteria or better adherence to treatment and follow-up among women. Despite the universally higher prevalence of KOA in women, most patients in our cohort were men, consistent with the broader trend that HTO is more frequently performed in male patients, possibly because women’s overall more severe symptoms often require TKA instead [32,44].

Regarding HTO type, no significant differences were found in terms of HTO success, with conflicting results between mean difference analyses and the univariate regression model, neither of which reached significance. The literature is also inconsistent on whether one technique is superior. In the meta-analysis by van Haeringen et al. (2023) [45], no differences were found in the risk of conversion to TKA according to HTO type, while Kim et al. (2017) [14] reported no differences at 5 years but a higher survival rate at 10 years for open-wedge procedures. Ultimately, we consider that the decision between techniques should account for other factors, such as the higher incidence of neurovascular complications associated with lateral closing-wedge HTO [13,46].

Finally, although 75% of patients requiring TKA in our study had a preoperative moderate to severe KOA stage, this was not significantly associated with treatment success. In the literature, however, this relationship is significant, with evidence pointing to higher baseline Kellgren–Lawrence grades being associated with worse HTO survival [16,34,40]. Specifically, Efe et al. (2011) [34] identified Kellgren–Lawrence grade > 2 as a threshold for increased risk. These findings are consistent with the rationale that in lower grades, cartilage deterioration is less advanced, and thus the load redistribution achieved through HTO may have greater clinical benefits.

As for disease progression, although we observed a tendency towards KOA stage stability in the first year after HTO, significant worsening was evident by the fifth year. This is consistent with the multifactorial pathophysiology of the disease, influenced not only by mechanical factors, which could be corrected by HTO, but also by biological and inflammatory processes. Such inevitable progression has been previously documented, with a tendency for progressive evolution of femoropatellar osteoarthritis to more advanced radiological grades after HTO [47]. However, it appears that although HTO does not halt the disease completely, it may slow its progression. In a recently published randomized trial, disease worsening was slower in patients treated with HTO compared to those managed conservatively, suggesting that HTO may positively influence the course of KOA [48].

Although the overall use of HTO has decreased in recent decades, its current role appears to be shifting toward a more selective application in carefully chosen patients. In particular, evidence shows an increase in HTO procedures among individuals under 50 years of age, suggesting that the technique has become increasingly specialized and tailored to specific clinical scenarios [32,44]. When contextualized with the existing literature, our results, albeit modest, are consistent with previous reports indicating that HTO should preferably be indicated in younger, active patients, as this group is more likely to achieve long-term survival of the procedure. Careful patient selection is therefore essential, as it may ultimately help optimize outcomes [34,36].

Our study has several limitations. It is retrospective in nature, and thus no formal sample-size calculation was performed prior to analysis. In addition, cases in which the planned mechanical correction was not achieved were excluded from the final cohort, as they did not meet predefined radiographic criteria for adequate alignment. While this approach ensured a homogeneous cohort in terms of technical execution, it may limit the generalizability of the findings to all HTO procedures performed in routine clinical practice. Furthermore, relevant clinical factors such as bone quality, concomitant procedures, axis alignment, degree of correction and function and quality of life scores were not included in this study and should be considered in future research. The cohort size was limited, and the uneven distribution of some variables, together with the low number of failure events, likely reduced statistical power for certain subgroups and multivariable analyses, which may have prevented the detection of associations of small-to-moderate magnitude. Moreover, the median follow-up time of 3.8 years is relatively short, and longer follow-ups would be more appropriate to confirm the durability of the observed outcomes. As a single-center observational series, residual confounding cannot be excluded. Inter-rater reliability for the radiographic assessments was not calculated. In addition, the number of patients available for radiographic assessment decreased over time, particularly at 5 years, which may affect the precision and stability of longitudinal progression analyses. Although missing radiographic data was due to patients not yet reaching later follow-up time points (administrative censoring), the complete-case structure at each time point may still influence interpretability. Specifically, the reduced sample size may affect the stability of the Bhapkar test used to assess marginal homogeneity over time and limit the robustness of long-term inferences. Consequently, these methodological constraints may reduce the external validity of our findings, and caution is warranted when extrapolating the results beyond the studied population. Nevertheless, we believe the data reflect real-world clinical practice and can inform hypothesis generation and the design of future prospective studies.

## 5. Conclusions

In conclusion, the Kaplan–Meier survival estimate for HTO was 76.7% at 5 years. Although age was associated with failure in univariate analysis, this relationship did not persist after multivariable adjustment, likely reflecting limited statistical power. Radiographic progression of KOA was evident at 5 years, reinforcing the concept that HTO may slow, but not halt, disease evolution.

## Figures and Tables

**Table 1:** Anthropometric, clinical and surgery parameters in the total sample of patients and stratified by treatment success. Data is presented as N (%) and mean ± SD. Standardized mean differences (SMDs) are provided to quantify the magnitude of between-group differences. Bold indicates statistical significance (*p* < 0.05).

Variable	Total (N = 63)	Success (N = 47)	Failure (N = 16)	SMDs	*p*-Value
Sex					
Male	45 (71.4%)	31 (66.0%)	14 (87.5%)	0.522	0.121
Female	18 (28.6%)	16 (34.0%)	2 (12.5%)		
Age (years)	53.3 ± 10.5	51.7 ± 11.3	58.1 ± 5.8	0.713	**0.006**
BMI (kg/m^2^)	29.6 ± 5.0	29.2 ± 5.0	30.7 ± 4.9	0.303	0.328
Comorbidities					
Hypertension	25 (39.7%)	18 (38.3%)	7 (43.8%)	0.110	0.700
Diabetes Mellitus	10 (15.9%)	7 (14.9%)	3 (18.8%)	0.084	0.705
Heart failure	6 (9.5%)	4 (8.5%)	2 (12.5%)	0.114	0.639
Respiratory failure	12 (19.0%)	10 (21.3%)	2 (12.5%)	0.221	0.714
Mental disorders	16 (25.4%)	13 (27.7%)	3 (18.8%)	0.204	0.740
HTO type					
Medial opening wedge	32 (50.8%)	25 (53.2%)	7 (43.8%)	0.187	0.514
Lateral closing wedge	31 (49.2%)	22 (46.8%)	9 (56.2%)		

**Table 2:** Univariate and multivariate Cox regression models for HTO survival time. Bold indicates statistical significance (*p* < 0.05). Reference categories: Male (sex), Medial opening wedge (HTO type), Doubtful (preoperative KOA stage).

	Univariate Model			Multivariate Model		
Variable	Hazard Ratio (HR)	95% CI	*p*-Value	Hazard Ratio (HR)	95% CI	*p*-Value
Sex						
Female	0.26	0.06–1.17	0.079	0.49	0.10–2.39	0.380
Age (years)	1.08	1.01–1.16	**0.033**	1.07	0.99–1.15	0.098
BMI (kg/m^2^)	1.05	0.96–1.16	0.272			
HTO type						
Lateral closing wedge	0.53	0.18–1.50	0.229			
Preoperative KOA stage						
Mild	3.38	0.35–33.0	0.295			
Moderate	5.11	0.63–41.7	0.128			
Severe	6.85	0.73–64.1	0.092			

**Table 3:** KOA stage at each follow-up in the total sample of patients and stratified by treatment success. Data are presented as fractions (n/N). In the overall sample, the denominator corresponds to the total number of patients at each follow-up time point. Patients who underwent TKA are indicated in the overall sample at each follow-up. “Not assessed” refers to patients who had not yet reached the follow-up time point and could therefore not be evaluated. In the success and failure subgroups, the denominator includes only patients evaluable for KOA stage at that specific follow-up, excluding those classified as “not assessed” and those who had undergone TKA.

Variable	Total	Success	Failure	*p*-Value
Preoperative KOA stage				
Doubtful	10/63	9/47	1/16	0.354
Mild	18/63	15/47	3/16	
Moderate	24/63	16/47	8/16	
Severe	11/63	7/47	4/16	
TKA	-	-	-	
Not assessed	-	-	-	
1-year follow-up KOA stage				
Doubtful	9/63	9/44	-	0.070
Mild	16/63	13/44	3/16	
Moderate	27/63	18/44	9/16	
Severe	8/63	4/44	4/16	
TKA	-	-	-	
Not assessed	3/63	-	-	
2-year follow-up KOA stage				
Doubtful	3/63	3/19	-	0.059
Mild	7/63	6/19	1/13	
Moderate	12/63	7/19	5/13	
Severe	10/63	3/19	7/13	
TKA	2/63	-	-	
Not assessed	29/63	-	-	
5-year follow-up KOA stage				
Doubtful	2/63	2/10	-	0.256
Mild	2/63	2/10	-	
Moderate	5/63	4/10	1/5	
Severe	6/63	2/10	4/5	
TKA	5/63	-	-	
Not assessed	43/63	-	-	

## Data Availability

The data presented in this study are available on request from the corresponding author, the data are not publicly available due to ethical restrictions.

## Supplementary Materials

The following supporting information can be downloaded at https://www.mdpi.com/article/10.3390/jfmk11010129/s1. Figure S1: Graphical assessment of Schoenfeld residuals for the multivariable Cox proportional hazards model. Each panel displays the scaled residuals for the covariates included in the model. No systematic trends were observed, supporting that the proportional hazards assumption was met both at the individual and global levels. Figure S2: Summary of Kaplan–Meier survival analysis for time to conversion to total knee arthroplasty (TKA) after high tibial osteotomy (HTO). Each panel shows the number at risk, number of events, percentage censored, and median survival time for the overall cohort, as well as stratified by sex, age group, HTO type, and preoperative knee osteoarthritis (KOA) stage.

## Abbreviations

The following abbreviations are used in this manuscript:

CI	Confidence Interval
HR	Hazard Ratio
HTO	High Tibial Osteotomy
KOA	Knee Osteoarthritis
SD	Standard Deviation
TKA	Total Knee Arthroplasty
