# Supplementary material for: High Tibial Osteotomy for Knee Osteoarthritis with Genu Varum: A Retrospective, Observational Study
Source: J Funct Morphol Kinesiol. 2026 Mar 23;11(1):129. doi: 10.3390/jfmk11010129 (PMC13027401; doi:10.3390/jfmk11010129)
Supplement: Supplementary file 1 [file jfmk-11-00129-s001.zip › Supplementary Figures.pdf]

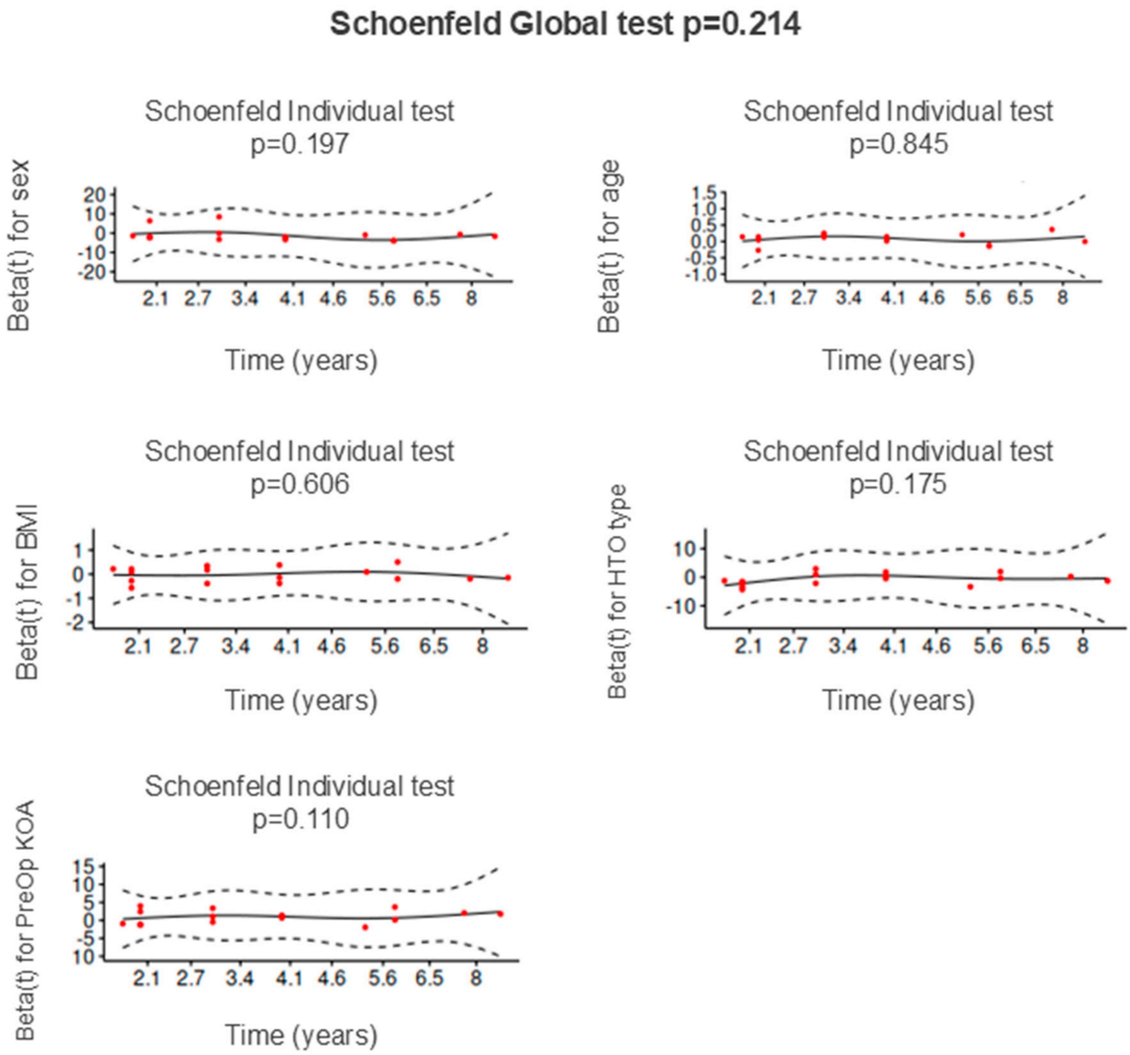

Figure S1. Graphical assessment of Schoenfeld residuals for the multivariable Cox proportional hazards model. Each panel displays the scaled residuals for the covariates included in the model. No systematic trends were observed, supporting that the proportional hazards assumption was met both at the individual and global levels.

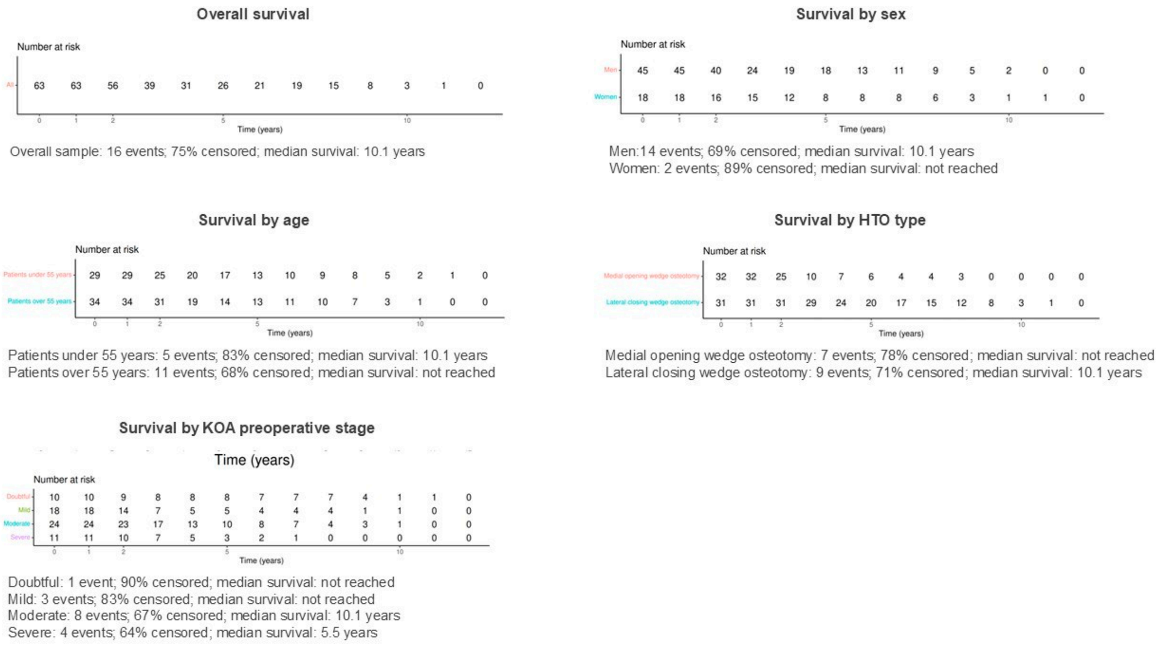

Figure S2. Summary of Kaplan–Meier survival analysis for time to conversion to total knee arthroplasty (TKA) after high tibial osteotomy (HTO). Each panel shows the number at risk, number of events, percentage censored, and median survival time for the overall cohort, as well as stratified by sex, age group, HTO type, and preoperative knee osteoarthritis (KOA) stage.
